# Human lifespan records are not remarkable but their durations are

**DOI:** 10.1371/journal.pone.0212345

**Published:** 2019-03-14

**Authors:** Anthony Medford, James W. Vaupel

**Affiliations:** Interdiscliplinary Center on Population Dynamics, University of Southern Denmark, Odense, Denmark; University of Jyväskylä, FINLAND

## Abstract

Has the maximum human lifespan been reached? The current record stands at 122 years, 164 days and has held for over 20 years and is more than four and three quarter years higher than the previous record. The value and persistence of this record have surprised some researchers, with some even questioning its veracity. There have been previous attempts in the literature to answer questions about how long this record might stand and whether it is truly exceptional but the focus has been mainly on the record ages, using *ad hoc* tools. This article contributes in two new ways. First we study lifespan records via the (inter-) record times and second we make use of specific tools from statistical Records Theory. We find that the occurrence of the present record was not surprising. We estimate around a 25% chance that the record would have survived until now and around a one in five chance that it will survive until 2050, demonstrating remarkable persistence.

## Introduction

Records are fascinating. Whether great sporting achievements, extreme weather events or even the absurd, records continue to intrigue. The Guiness Book of Records remains the best-selling copyrighted book of all time. In particular, demographic records are of great interest.

There has been much debate—even controversy—surrounding the length of human lifespan and where it might be heading. With the steady rise in life expectancy over the past 175 years, interest in human longevity has grown. Jeanne Calment died on 4 August 1997 aged 122 years, 164 days, with the dual distinctions of being the oldest verified human and the only person to attain a lifespan of at least 120 years.

Many have wondered about the durability of her record and when, or if ever, it might be broken. The chances of her record being broken offer insights into the plausibility of lifespan limits, with a higher chance of the record being surpassed making the argument for a limit less tenable. Recently, [1] expored these and similar questions. However their approach was *ad hoc*. They concluded that the existing record is not surprising and could endure for decades. Given that there is a substantial and well developed theory of records, why not use it directly to study lifespan records? Our aim is to fill this gap in the literature.

Records theory, not surprisingly, is closely related to extreme value theory but the two are not equivalent. All records are extreme values but the reverse is not true. Previous applications of extreme value theory to the study of lifespans addressed the question of whether there are limits to human lifespan. Among these are the work of [2–5]. In this article we do not directly engage this question.

The earliest developments of the theory of records go back to [6]. Since then there have been numerous and substantial contributions and the works of [7–9] and [10] among others provide extensive reviews. This classical theory assumes independent, identically distributed (iid) records and is not realistic for many situations. [11] was among the first to tackle the issue by considering geometrically increasing populations and since then other non-iid record schemes have been introduced by [12, 13] and [14] among others.

Unfortunately, the term record has often been misused in the demographic literature. A record is not merely an extreme value but it is the most extreme value observed up to the present time and it is not uncommon to see annual maxima being imprecisely labelled records [15, 16]. Later on we introduce a more formal definition. Concerning human lifespan, there are a number of studies of the annual maxima e.g. [17, 18]. Conversely, very few analyses have been done with lifespan data using the formal definition of records.

There are two approaches to the study of records: either from the usual perspective of the record values or the perspective of the times at which records occur. Previous work with lifespan records has taken the record value approach [1]. In this article we view the problem from the perspective of the record times. We examine the (inter-) record times set by the longest lived individuals and only consider supercentenarians—those who have lived to the age of at least 110 years.

To the best of our knowledge this is the first study to apply the theory of records to human lifespan records. The structure of this article is as follows. We first present the data and methodology used. Then we present the results, including historical probabilities of record breaking and a probability distribution for the time between records. Finally, we offer some discussion and conclude.

## Data and methods

The Gerontological Research Group (GRG) maintains a list of verified supercentenarians. According to the GRG’s website, “Verified supercentenarians have multiple documents throughout their life course to prove their age, including, at minimum, one early-life document; one mid-life document; and one late-life document”. We obtained a dataset of verified supercentenarians who died between 1899 and 2014 inclusive, totalling 1943 lives.

In this article ‘lifespan’ refers to completed life and is defined to be the exact length of time between the birth and death of an individual, so we are concerned with the record ages at death. On May 12, 1990 Jeanne Calment attained age 115.2 years and became the oldest verified human; she died over 7 years later. The age of the oldest person alive, though also an interesting record, is not our focus and will not be discussed further.

The word ‘record’ also has a specific meaning and a precise mathematical definition as given below. A lifespan record at some given point in time is defined to be the highest age at death observed from among all prior deaths to date.

The theory of records is concerned with values that are strictly larger (or smaller) than all previous values. Let *X*_1_…, *X*_*n*_ be a series of independent, identically distributed random variables with *X*_*t*_ having a continuous distribution. We say that *X*_*t*_ is a record if and only if it is the largest among all *X*_*t*_ that have been observed up to time *t*. More precisely, *X*_*t*_ is an upper record if *X*_*t*_ > max(*X*_1_…, *X*_*t*−1_) for *t* ≥ 2. Lower records are where *X*_*t*_ < min(*X*_1_…, *X*_*t*−1_) for *t* ≥ 2. Since max(*X*_1_…, *X*_*n*_) is equivalent to min(−*X*_1_…, −*X*_*n*_), our focus will be on upper records and henceforth ‘record’ shall be taken to mean upper record. Selected details of this basic theory for the iid scenario are given in S1 Appendix.

### Growing populations

**Fig 1:**
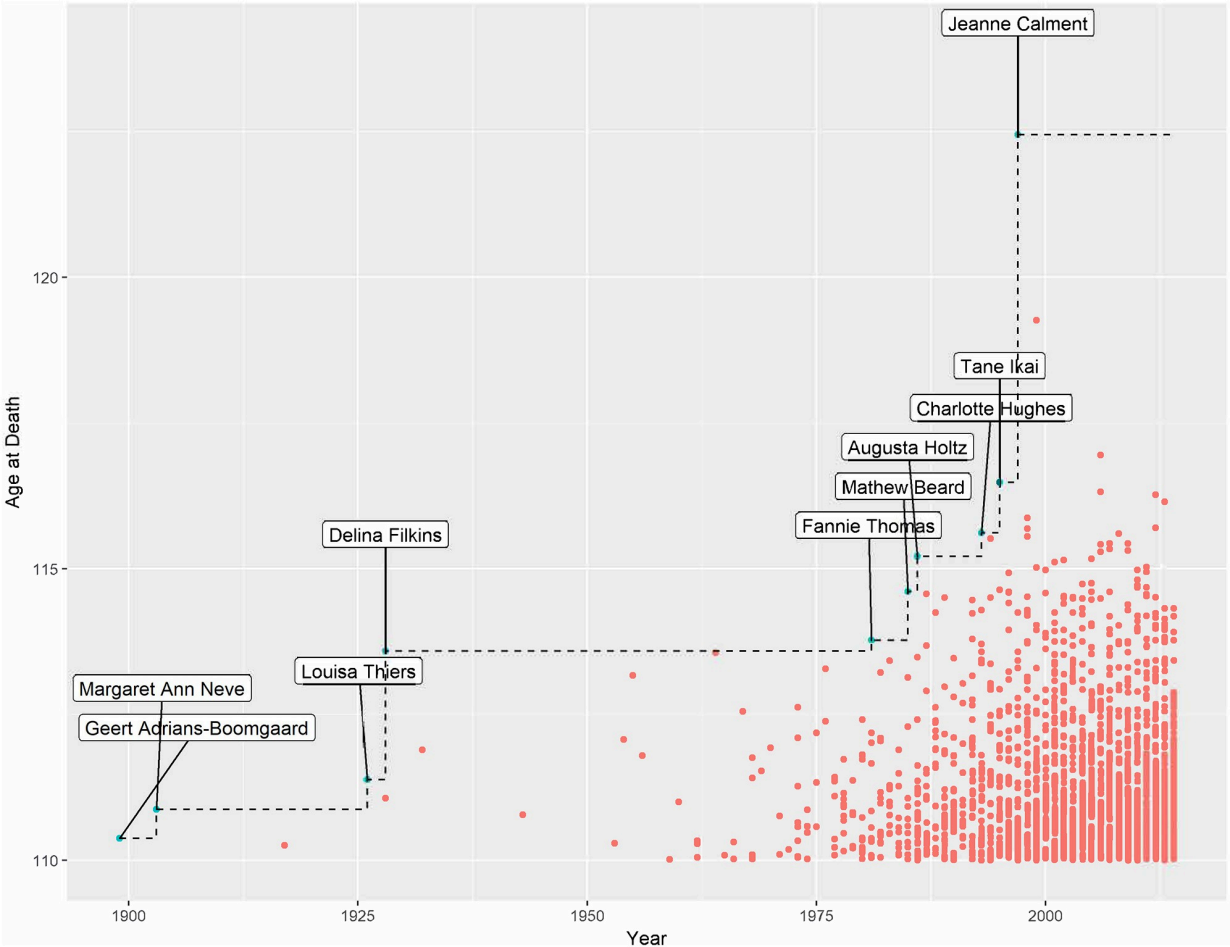
Supercentenarian lifespans and recordholders. Verified supercentenarians who died between 1899 and 2014. Each dot represents a death with the blue dots indicating a record setting age.

Given the increasing numbers attaining high ages, a realistic model should reflect this growing population. Between 1899 and 2014 the number of supercentenarians in the dataset increased at an annualized rate of 6.7%. To account for this we will consider the model of [11] which assumes a geometrically increasing population. Motivated by the increasing frequency of record breaking in the Olympic games, this model attempted to account for the influence of population size on record breaking. Fig 1 suggests that geometric increase is a reasonable assumption. The population could also be said to be exponentially increasing as well since this is just the continuous version of geometric increase. The exponential growth assumption can be tested in a straightforward way. If the exponential assumption is true then a linear regression of the natural logarithm of the population size against year should fit well. This test was performed, using data from 1970 which is roughly when we started to regularly observe more than one or two supercentenarians per year. The regression gave an *R*^2^ of about 92%, indicating an excellent fit and strong evidence for the exponential assumption. [19] generalized this model to the so called *F*^*α*^ model.

Our particular model is formulated as follows. Since we are interested in changes over time, assume that at each time point *t*, an *n*_*t*_ sized population is available. Specifically, assume that the random variable *X*_*t*_ is the maximum of an increasing number *n*_*t*_ of iid random variables. That is $X_{t}=\text{max}\left\{X_{t,1},X_{t,2},\ldots ,X_{t,n_{t}}\right\}$ where *X*_*t*,*i*_, *t* = 1899, …, 2014; *i* = 1, 2, …, *n*_*t*_ is a doubly indexed sequence of iid random variables with continuous distribution function $F_{X_{t}}\left(x\right)$ and *n*_*t*_ is the population size in year *t*.

Then, *X*_*t*_ is a sequence of independent random variables with $F_{X_{t}}\left(x\right)={\left[F\left(x\right)\right]}^{n_{t}},t=1899,\ldots ,2014$. For a geometrically increasing population, *n*_*t*_ grows at the rate say *θ* per year such that *n*_*t*_ = *n*_1_(1 + *θ*)^*t*−1^. The limiting distribution of the inter-record time converges to a Geometric distribution with success probability *p* = *θ*/(1 + *θ*). Detailed proofs can be found in [11].

## Results

**Fig 2:**
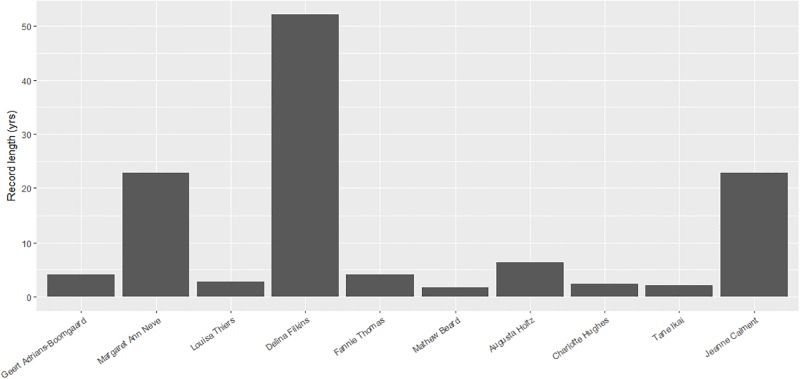
Record lengths. The length of time that each record lasted i.e. the inter-record time.

Fig 1 presents the data. Note the rapid increase in supercentenarians since the mid-1970s. The first verified supercentenarian was the Dutchman Geert Adrians-Boomgaard who died in the Netherlands in 1899. By definition, the first entry is the first record and is known as the trivial record. All other records are non-trivial. Subsequently, there have been 9 other record holders including Jeanne Calment who is the current record holder. The length of time that each record lasted is shown in Fig 2. The median inter-record time is around 4.1 years and the mean time is around 11.9 years (s.e. = 5.1 years).

There were three records that lasted for more than 20 years: Jeanne Calment (20.7 years and counting), Margaret Ann Neve (22.9 years) and Delina Filkins with the longest lasting record at just over 52 years. Given that Calment’s record is about 20.7 years old, her record may endure for some time still.

Our statistical framework enables us to perform two key actions concerning lifespan records:

- Calculate the probability of record breaking during a given year
- Derive a probability distribution for the inter-record times (which in turn allows the calculation of further items of interest)

### Probability of record breaking

**Fig 3:**
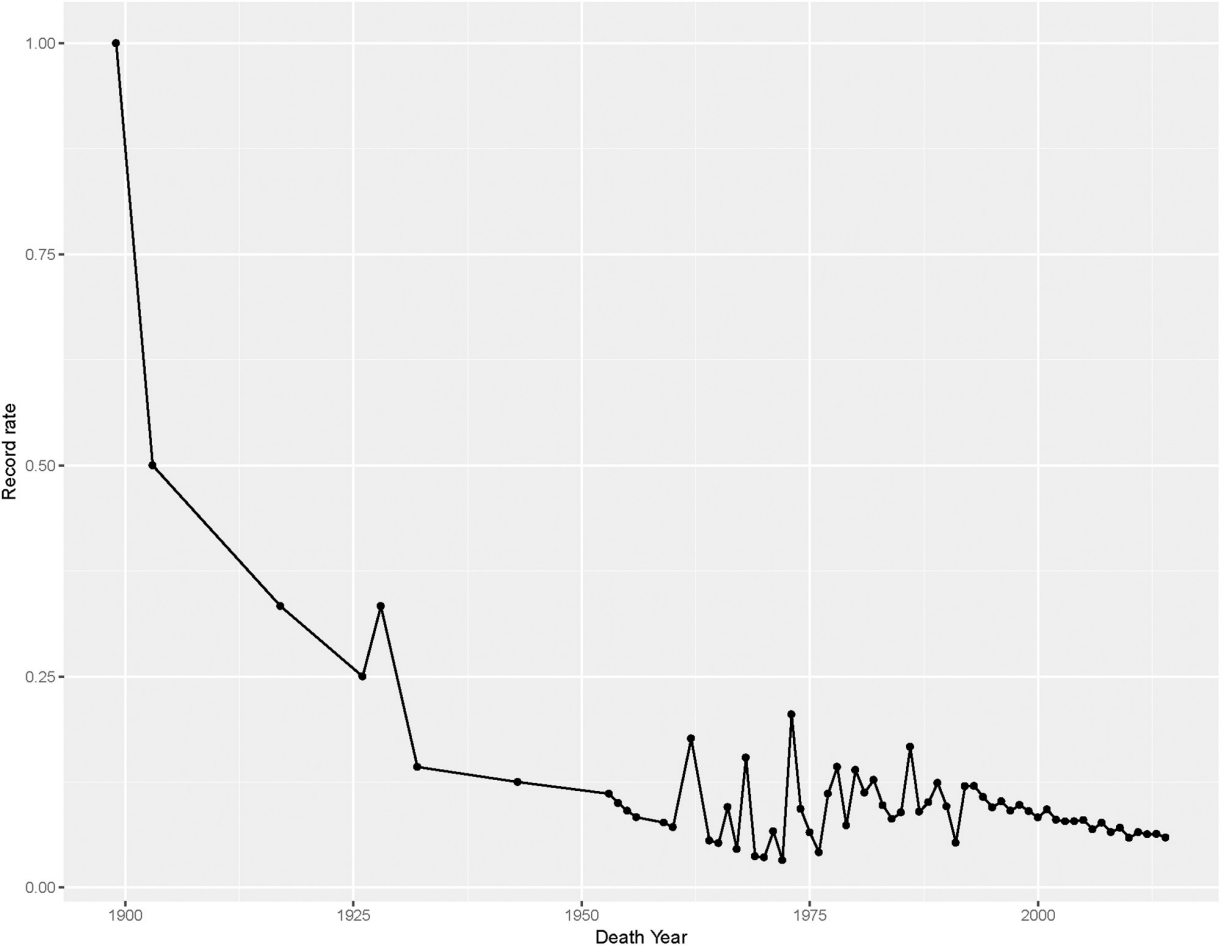
Historical record rates. Historical probabilities that a supercentenarian death would result in a new record. Each dot represents the record rate for that year. The connecting lines are added to assist in visualisation of the trend.

Let the probability of observing a record during year *t* be *P*_*t*_, also known as the record rate for year *t*. The record rate in year *t* is *n*_*t*_/(*n*_1_ + *n*_2_ + ⋯ + *n*_*t*_). Further details on this can be seen in S1 Appendix. Fig 3 presents the historical record rates (see also S2 Appendix). What is remarkable is the stability of the record rate over time, particularly since the mid 1970s when the number of supercentenarians began to increase exponentially. From a records theory perspective this is not surprising because the record rate for exponentially increasing populations is constant. In fact, as the size of the supercentenarian population grows large, the record rate *P*_*t*_ converges to 1 − exp(−*c*) where *c* in the exponential rate of increase [20]. The long term record rate for this population is 6.3%. This means that if the number of supercentenarians continues to grow exponentially, eventually the probability that a supercentenarian death during a given year will result in a new record in that year will stabilise and remain at a level of around 6.3%. That is, *c* = *log*(1 + *θ*), where *θ* is the geometric rate of increase.

If we make the assumption that the supercentenarian population continues to grow at the current rate, the probability of observing a new record in the year 2020 would be around 6.0% but rise only slightly to 6.3% in 2050. In fact, the record rate is projected to reach its limiting value around 2067. Suppose that the growth rate is 25% higher than currently, then the record rate in 2020 and 2050 would then be 6.5% and 7.7% respectively. Similarly, if the assumed growth rate is 25% lower than currently, the record rates would instead be 5.6% and 5.0% respectively. Suppose that the supercentenarian population grows at the current rate of the overall population (1.3%) then the record rate in 2050 would be 2.5%. So the record rate does not seem to be especially sensitive to reasonable changes in the population growth rate but of course, it could be sensitive to dramatic changes in the growth rate.

Given the probability of a broken record, it is straightforward to calculate the expected number of records observed up to some time (S2 Appendix). Currently we have observed 10 (9 non-trivial) lifespan records at the end of 2014. Our model suggests that we would expect around 8 records (standard deviation 2.4). This matches closely what has been observed, and provides reassurance in the validity of the model.

### Distribution of inter-record times

How long might the current record persist? First we need an estimate of the growth rate of the supercentenarian population. Between 1899 and 2014 the world’s population increased from roughly 1.6 billion to 7.5 billion people, corresponding to an annual growth rate of *θ* = 1.3%. However, during the same period the supercentenarian population experienced a growth rate of *θ* = 6.8%, or 5 times faster than the general population.

As the population size grows, the inter-record time converges in distribution to a Geometric random variable with parameter *p* = .068/1.068 = 0.063, the success probability. So the expected waiting time between successive records is 1/.063 or approximately 15.8 years. This is longer than the observed mean waiting time of 10.9 years between records. This is not surprising, since the growth rate in population is only partly responsible for the accumulation of the oldest old. In addition to larger numbers, the populations have been experiencing fundamental improvements in mortality.

What is the probability that the record has not yet been broken? Given that roughly 20.7 years have passed since the last lifespan record breaking, we need the probability that the time to the next record is greater than 20.7 years. More precisely, we calculate the probability that the record has not been broken by the 21st year (the Geometric distribution is discrete). This is found to be about 25%. In other words, there was a 75% chance of observing a new record in the time since the last one so it is somewhat surprising that the record still holds. However, 20.7 years is still quite low when compared to the most durable record, which lasted 52 years.

Similarly, we can calculate the probability of observing a new record by the year 2050. This is the conditional probability that the inter-record time is less than or equal to 53 years given that no record has been observed up to year 21. This probability is approximately 82%.

## Discussion and conclusions

We presented a novel way to study trends in human lifespan. We analysed the record breaking times of the human lifespan and the lengths of the inter-record times. In the period since the mid 1970s when the number of supercentenarians increased substantially we observe that the annual probability of observing a new record has been remarkably stable over time.

An interesting feature of our approach is that the results are achieved without the need to assume any particular model for the underlying population mortality. This is an important advantage, particularly at the highest ages where data are sparse and it is easy to mis-estimate correct mortality trajectories. The likely trajectory of mortality at these ages remains an area of intense and often controversial debate.

One key limitation of our analysis is the data. Given that there is a lag between the occurrence of supercentenarians and updating the database due to the time taken to verify and sometimes even discover cases, the number of supercentenarians listed can be less than the true number. This biases the value of the population growth rate downwards. There is also age ascertainment bias in how the GRG collects its data as they focus on the oldest cases. Given that our data window ended in 2014, over three years ago, this issue is mitigated somewhat because sufficient time has passed to enable the outstanding cases to be added to the database. There is also the issue of age exaggeration, where persons tend to overstate their ages but this issue is mostly dealt with by the verification process.

Viewed within the context of the overall evolution of annual record rates and inter-record times it is very difficult to say whether the occurrence of Jeanne Calment’s record was surprising or not. The record rates in the years leading up to the emergence of her record were not extraordinary and the inter-record times of the records adjacent to hers were not very different from what had been observed previously. However, we can argue that her record has been remarkably durable especially since the supercentenarian population has been rapidly increasing in size and improving with respect to underlying mortality over the last few decades. We found there was only a one in four chance that the her record would have survived until now—yet it still persists. According to the Gerontology Research Group, the current oldest living person in the world, Kane Tanaka, turned 115 years old on January 2, 2018. As at July 31, 2018 she is aged 115 years and 210 days, making the record of Jeanne Calment safe for at least another 6 years and 319 days days from this date. Of course, this can happen only if Kane Tanaka survives that time. Even though this is possible, the probability of this happening is very small. In fact, assuming a fixed annual death probability of 0.5 [21] this probability is approximately 0.0085, so Calment’s record will likely survive for some time yet.

Looking ahead, there is almost a one in five chance that Calment’s record would still hold in the year 2050. This is rather impressive and demonstrates that her record is surprising not in terms of its timing but in terms of its duration. In the theory of records the next record occurs in finite time with probability one. In other words, “all records are meant to be broken”. This may be no different for the length of human lifespan, except that we do not know when.

## Supporting information

S1 Appendix Record models. Basic outline of iid and increasing population models.
(PDF)

S2 Appendix Historical record rates. Record rates and other related record statistics.
(PDF)

S1 Dataset Data used to fit model. (XLSX)
