# Supplementary material for: Human lifespan records are not remarkable but their durations are
Source: PLoS One. 2019 Mar 14;14(3):e0212345. doi: 10.1371/journal.pone.0212345 (PMC6417653; doi:10.1371/journal.pone.0212345)
Supplement: S1 Appendix — Basic outline of iid and increasing population models. (PDF) [file pone.0212345.s001.pdf]

Appendix 1: Record Models

Classic Model: iid population

Given iid $X_1 \ldots, X_n$, what is the probability that the value of X_n will be a record? All variables of $X_1 \ldots, X_n$ are mutually different with probability 1. They can be ordered in $n!$ ways, and all orderings have the same probability X_n is a record if it is the largest of all observed and there are $(n-1)!$ ways of ordering the remaining $n-1$ variables. The probability that X_n will be a record, P_n, say is then

$$P_n = \frac{(n-1)!}{n!} = \frac{1}{n}$$

This is sometimes referred to as the record rate. For ease in computation we introduce the indicator variables $Y_1 \ldots, Y_n$ where $i = 1 \ldots n$ and let

$$Y_i = \begin{cases} 1 & \text{if } X_i \text{ is a record} \\ 0 & \text{if } X_i \text{ is not a record} \end{cases}$$

The total number of records in the series $X_1 \ldots, X_n$ is

$$R_n = Y_1 + \cdots + Y_n,$$

so that the expected number of records up to a time n is

$$E(R_n) = \sum_1^n Y_i = E(Y_1) + \cdots + E(Y_n) = 1 + 1/2 + \cdots + 1/n$$

F^α Model: increasing population

The F^α model is defined such that the underlying independent random variables $X_1, X_2, \ldots$ are no longer constrained to be identically distributed and have distribution functions $F_1, F_2, \ldots$ so that $F_n = F^{\alpha(n)}, \alpha(n) > 0$. The case where $\alpha(n) = 1$ for all n is the classical model and geometrically increasing $\alpha(n)$ is the Yang model.

In the F^α model the record indicators Y_i are still independent and many of the results from the iid case carry over in a comparable way. The record rate is then

$$P_n = Pr(Y_n = 1) = 1 - Pr(Y_n = 0) = \frac{\alpha(n)}{\alpha(1) + \cdots + \alpha(n)}, \qquad n = 1, 2, \ldots$$

so that the expected number of records up to a time n is

$$E(R_n) = \sum_{j=1}^n \frac{\alpha(j)}{\alpha(1) + \cdots + \alpha(j)}$$
