# Supplementary material for: Human lifespan records are not remarkable but their durations are
Source: PLoS One. 2019 Mar 14;14(3):e0212345. doi: 10.1371/journal.pone.0212345 (PMC6417653; doi:10.1371/journal.pone.0212345)
Supplement: S2 Appendix — Record rates and other related record statistics. (PDF) [file pone.0212345.s002.pdf]

Appendix 2: Historical record rates

	Year	Deaths	Record rate	Expected no. records	Std. deviation
1	1899	1	1.000	1.00	0.00
2	1903	1	0.500	1.50	0.50
3	1917	1	0.333	1.83	0.69
4	1926	1	0.250	2.08	0.81
5	1928	2	0.333	2.42	0.94
6	1932	1	0.143	2.56	1.00
7	1943	1	0.125	2.68	1.06
8	1953	1	0.111	2.80	1.10
9	1954	1	0.100	2.90	1.14
10	1955	1	0.091	2.99	1.18
11	1956	1	0.083	3.07	1.21
12	1959	1	0.077	3.15	1.24
13	1960	1	0.071	3.22	1.26
14	1962	3	0.176	3.39	1.32
15	1964	1	0.056	3.45	1.34
16	1965	1	0.053	3.50	1.36
17	1966	2	0.095	3.60	1.39
18	1967	1	0.045	3.64	1.41
19	1968	4	0.154	3.80	1.45
20	1969	1	0.037	3.83	1.46
21	1970	1	0.036	3.87	1.48
22	1971	2	0.067	3.94	1.50
23	1972	1	0.032	3.97	1.51
24	1973	8	0.205	4.17	1.56
25	1974	4	0.093	4.27	1.59
26	1975	3	0.065	4.33	1.61
27	1976	2	0.042	4.37	1.62
28	1977	6	0.111	4.49	1.65
29	1978	9	0.143	4.63	1.69
30	1979	5	0.074	4.70	1.71
31	1980	11	0.139	4.84	1.74
32	1981	10	0.112	4.95	1.77
33	1982	13	0.127	5.08	1.80
34	1983	11	0.097	5.18	1.82
35	1984	10	0.081	5.26	1.84
36	1985	12	0.089	5.35	1.87
37	1986	27	0.167	5.51	1.90
38	1987	16	0.090	5.60	1.92
39	1988	20	0.101	5.71	1.95
40	1989	28	0.124	5.83	1.98
41	1990	24	0.096	5.93	2.00
42	1991	14	0.053	5.98	2.01
43	1992	36	0.120	6.10	2.04

44	1993	41	0.120	6.22	2.06
45	1994	41	0.107	6.33	2.08
46	1995	40	0.095	6.42	2.11
47	1996	48	0.102	6.52	2.13
48	1997	47	0.091	6.61	2.15
49	1998	56	0.098	6.71	2.17
50	1999	57	0.090	6.80	2.19
51	2000	57	0.083	6.89	2.20
52	2001	70	0.092	6.98	2.22
53	2002	66	0.080	7.06	2.24
54	2003	70	0.078	7.14	2.25
55	2004	76	0.078	7.21	2.27
56	2005	84	0.080	7.29	2.29
57	2006	78	0.069	7.36	2.30
58	2007	94	0.077	7.44	2.32
59	2008	86	0.066	7.51	2.33
60	2009	100	0.071	7.58	2.34
61	2010	88	0.059	7.64	2.35
62	2011	105	0.065	7.70	2.37
63	2012	108	0.063	7.76	2.38
64	2013	116	0.063	7.83	2.39
65	2014	115	0.059	7.89	2.40

Table 1: Annual record rates along with expected number of records and standard deviation of expected number of records.
